# Addition of Metabolic Syndrome to Albuminuria Provides a New Risk Stratification Model for Diabetic Kidney Disease Progression in Elderly Patients

**DOI:** 10.1038/s41598-020-63967-9

**Published:** 2020-04-22

**Authors:** Hong-Mou Shih, Shih-Ming Chuang, Chun-Chuan Lee, Sung-Chen Liu, Ming-Chieh Tsai

**Affiliations:** 1 Division of Nephrology, Department of Internal Medicine, Mackay Memorial Hospital, Taipei, Taiwan; 2 Division of Endocrinology and Metabolism, Department of Internal Medicine, Mackay Memorial Hospital, Taipei, Taiwan; 3 Mackay Junior College of Medicine, Nursing, and Management, Taipei, Taiwan; 4 Graduate Institute of Physiology, College of Medicine, National Taiwan University, Taipei, Taiwan

**Keywords:** Ageing, Metabolic syndrome, Diabetes complications, Chronic kidney disease

## Abstract

Elderly patients with type 2 diabetes (T2DM) are more prone to developing diabetic kidney disease (DKD). Patients with DKD can develop albuminuria, and some studies have suggested an association between metabolic syndrome and albuminuria. The prevalence of both metabolic syndrome and albuminuria increases with age. We evaluated the association of these risk factors with worsening renal function and albuminuria progression in 460 T2DM patients with a mean age of 72 years. During the 5-year follow-up period, progression of albuminuria and worsening of renal function were observed in 97 (21.2%) and 23 (5.1%) patients, respectively. After adjusting for confounding factors, the group with metabolic syndrome had a higher multivariable-adjusted hazard ratio (HR) for worsening renal function (P = 0.038) and albuminuria progression (P = 0.039) than the group without metabolic syndrome. When patients were divided into four groups according to the presence of metabolic syndrome and/or albuminuria, the HR gradually increased. The group with both albuminuria and metabolic syndrome exhibited the highest cumulative incidence of worsening renal function (P = 0.003). When we redefined metabolic syndrome to exclude the blood pressure (BP) component, similar results were obtained. We concluded that the presence of metabolic syndrome independently predicts the progression of renal disease in elderly patients with T2DM. The use of both metabolic syndrome and albuminuria provides a better risk stratification model for DKD progression than albuminuria alone.

## Introduction

The prevalence of diabetes and diabetic kidney disease (DKD) continues to increase worldwide. Type 2 diabetes (T2DM) is the leading cause of chronic kidney disease (CKD) and end-stage renal disease (ESRD)^1,2^. DKD is primarily evaluated and monitored by the assessment of kidney function, usually based on estimated glomerular filtration rate (eGFR) and albuminuria, which is an established risk marker for renal function^1^. The United Kingdom Prospective Diabetes Study (UKPDS) 15-year observational cohort study found that nearly 40% of UKPDS patients developed albuminuria and nearly 30% of patients developed renal function impairment. This analysis revealed that the risk factors for the development of albuminuria and renal function impairment were different, which may reflect the distinct pathological processes of these two renal outcomes in patients with T2DM^3^. Albuminuria is thought to be an independent predictor of incident cardiovascular disease. Some observational studies found that a higher rate of urinary albumin excretion was associated with a higher incidence of cardiovascular morbidity and mortality^4,5^. Other studies have suggested that elevated levels of microalbuminuria strongly predict the development of DKD, but whether microalbuminuria is a predictor of DKD progression remains controversial^6–8^. Elderly patients with T2DM are prone to developing DKD due to the accelerated decline in kidney function and increased albuminuria with age, and DKD is more likely to be underestimated in elderly T2DM patients^9^.

Metabolic syndrome is strongly correlated with T2DM because of the relationship between obesity and insulin resistance^10^. There is increasing evidence that metabolic syndrome is associated with incident and prevalent CKD^11,12^. The third National Health and Nutrition Examination Survey (NHANES III) demonstrated that metabolic syndrome is independently associated with microalbuminuria, and some authors believe that microalbuminuria may also be a component of metabolic syndrome^13^. Although small-scale studies found that metabolic syndrome can predict renal function deterioration in non-diabetic patients with early CKD, the impact of metabolic syndrome on renal function deterioration in diabetic patients is currently inconclusive^14,15^. The prevalence of metabolic syndrome also increased with age in the NHANES III cohort, from 6.7% in participants aged 20 to 29 years to 43.5% in subjects over 60 years of age^16^. There is a well-known association between metabolic syndrome and the development of CKD in the elderly; however, this is mainly for people without diabetes^11,17^. Compared to other costly novel risk markers, identification of metabolic syndrome as a risk factor may be a cost-effective marker that is also convenient for clinicians. Considering that the prevalence of metabolic syndrome and albuminuria increases with age, and both of these risk factors have been associated with diabetes, we focused on elderly patients with T2DM aged 65 years or older. It is not known whether metabolic syndrome or albuminuria promotes renal disease progression in these patients, and only few studies have evaluated the combined effect of metabolic syndrome and albuminuria on renal outcomes. We hypothesized that the presence of both factors may promote the worsening of renal function or albuminuria progression in elderly subjects with T2DM.

## Materials and Methods

We recruited 812 subjects with T2DM, aged 65 to 91 years of age, who were followed up for diabetes management and evaluation of diabetes complications at the Endocrine outpatient department of the Taipei branch of Mackay Memorial Hospital from October 17, 2013 to February 7, 2015. For all 812 patients enrolled in the study, a routine medical history and physical examination was performed at the outpatient clinic, and blood samples were taken every two to three months. We excluded patients with macroalbuminuria or estimated glomerular filtration rate (eGFR) <15 ml/min/1.73 m^2^ (n = 128). Patients who met the criteria were followed until January 2018. A total of 224 patients were excluded due to incomplete data for at least 1 year during the follow-up period. We also excluded 42 patients with cardiovascular disease (n = 14), cerebrovascular disease (n = 18), malignant disease (n = 10) or sepsis (n = 4), retaining a total of 460 patients. The study protocols were approved by the Institutional Ethics Committee of Mackay Memorial Hospital (18MMHIS104e). All participants provided written informed consent to participate in the study. All methods were carried out in accordance with relevant guidelines and regulations (Declaration of Helsinki).

We recorded data obtained during routine physical examinations, including age, blood pressure (BP) and anthropometry measurements such as waist circumference and body mass index (BMI). The patients were required to sit for 5 minutes before trained nurses measured BP twice. The reported BP was calculated by averaging the two readings. We also recorded participants’ medical history (smoking and alcohol consumption) and diabetes duration based on medical records.

Laboratory tests using venous blood samples collected after an overnight fast for 12 hours included fasting plasma glucose (FPG), postprandial plasma glucose (PPG), glycated hemoglobin (HbA1c), total cholesterol (TC), triglycerides (TG), low-density lipoprotein cholesterol (LDL-C), high-density lipoprotein cholesterol (HDL-C), liver enzymes, serum creatinine, eGFR and urinary albumin-creatinine ratio (ACR).

### Measurement of urinary albumin-creatinine ratio and estimated glomerular filtration rate

The definition of urinary ACR is based on urinary albumin (measured by immunoturbidimetry) corrected for urinary creatinine levels. Levels of urinary albumin were described as follows: normoalbuminuria, ACR < 30 mg/g Cr; microalbuminuria, ACR: 30–299 mg/g Cr; and macroalbuminuria, ACR > 300 mg/g Cr. Acute febrile illness, excessive exercise, urinary tract infections, persistent hyperglycemia, and hypertensive crisis were all excluded due to the resultant transient increase in urinary albumin excretion^18^.

### Definition of metabolic syndrome

Metabolic syndrome was defined according to the National Cholesterol Education Program Adult Treatment Panel III (NCEP-ATPIII) and modified criteria including the waist circumference cutoff for Asian populations^19^ and three of the following abnormalities: (1) central obesity or abdominal obesity with a waist circumference of at least 80 cm in females and 90 cm in males; (2) fasting TG level of at least 150 mg/dL; (3) HDL-C level below 40 mg/dL in males and 50 mg/dL in females; (4) blood pressure of at least 130/85 mmHg or previously diagnosed hypertension; and (5) fasting blood glucose of at least 110 mg/dL or previously diagnosed diabetes.

All patients enrolled in our study had diabetes, so they all met the blood glucose component criterion. Thus, as long as two or more of the other components were present, patients were considered to have metabolic syndrome. In addition, patients taking antihypertensive drugs were recorded as satisfying the blood pressure criterion and patients using lipid-lowering drugs including fibrates and/or statins were recorded as fulfilling the lipid criterion.

### Outcome

In this study, two CKD event endpoints were defined: progression of albuminuria and worsening renal function. Because we obtained a random urine sample and measured urine albumin for each patient at baseline, we were able to use the change in ACR to define the progression of albuminuria according to the transition from normo- to microalbuminuria or micro- to macroalbuminuria. In order to avoid misclassification, only those patients who had at least two consecutive ACRs per year and were followed up for more than 1 year were included in the analysis. The end point for worsening renal function was defined as a 50% reduction in eGFR, doubling of serum creatinine or ESRD. Each subject who participated in the study had at least one serum creatinine or eGFR measurement per year, which could be compared to baseline creatinine or eGFR values.

All protocols were approved by the Institutional Ethics Committee of Mackay Memorial Hospital (18MMHIS104e).

### Statistical analysis

Statistical analysis was performed using IBM SPSS release 21.0 (IBM, Armonk, NY, USA). Baseline data for the study participants are expressed as a percentage or mean ± standard deviation (SD) for normally distributed variables or median (interquartile range) for skewed variables.

Differences between the four groups (normoalbuminuria or microalbuminuria with or without metabolic syndrome) were analyzed by one-way analysis of variance (ANOVA) followed by Bonferroni’s post hoc test for normally distributed values. A chi-square test was used to analyze the differences in baseline characteristics.

The cumulative incidences of primary end points, including progression of albuminuria and worsening renal function, were estimated with the Kaplan–Meier method and log-rank test. Univariable and multivariable Cox regression analyses were performed to assess the risk estimates for reaching each end point. Results are presented as the hazard ratio (HR) and 95% confidence interval (CI). The following variables were incorporated as covariates in multivariable Cox regression analysis: gender, obesity (BMI > 28 kg/m^2^), diabetes duration, HbA1c, hypertension, dyslipidemia, baseline eGFR, use of renin-angiotensin system inhibitors (ACEI or ARB), and CKD stage 3–4 (15 ≦ eGFR <60) at baseline for the endpoint of albuminuria progression; diabetes duration and HbA1c for the endpoint of worsening renal function. A two-tailed P-value < 0.05 was considered statistically significant.

## Results

### Baseline patient characteristics

**Figure 1:**
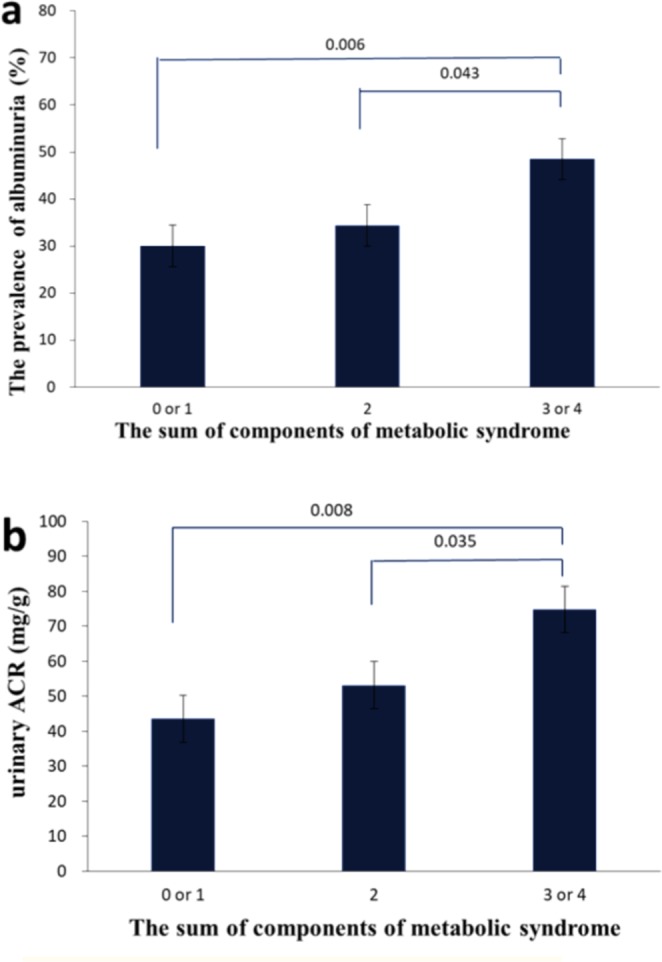
Prevalence of microalbuminuria (**a**) and urinary ACR (**b**) according to the number of metabolic syndrome components. ACR albumin-creatinine ratio.

The baseline clinical and biochemical characteristics with associated endpoints of 460 T2DM patients who fulfilled the study criteria and had an adequate follow-up period are shown in Table 1. The mean age of these patients was 72 years, and 196 patients (42.6%) were men. Of all the patients, 306 (66.5%) had metabolic syndrome. The UACR and prevalence of microalbuminuria increased with an increasing number of metabolic syndrome components (Fig. 1). We categorized all patients into four groups based on the presence of normoalbuminuria or microalbuminuria with or without metabolic syndrome: 116 patients with normoalbuminuria without metabolic syndrome, 204 patients with normoalbuminuria and metabolic syndrome, 38 patients with microalbuminuria without metabolic syndrome, and 102 patients with microalbuminuria and metabolic syndrome. There were no significant differences in gender, smoking, FPG, TC, LDL, diastolic blood pressure (DBP), glutamic pyruvic transaminase (GPT), lipid-lowering medication, and ACEI or ARB medications between the four groups. Patient BMI was higher in the group with metabolic syndrome than in the group without metabolic syndrome. In the microalbuminuria group, patients were older and had a longer diabetes duration, higher HbA1c and a greater prevalence of CKD stage 3–4. In addition, there was a small but statistically significant difference in systolic blood pressure (SBP).

**Table 1:** Clinical characteristics and laboratory data of study subjects with type 2 diabetes according to either microalbuminuria or metabolic syndrome.

	Normoalbuminuria		Microalbuminuria		P value
	Without metabolic syndrome (N = 116)	With metabolic syndrome (N = 204)	Without metabolic syndrome (N = 38)	With metabolic syndrome (N = 102)	
Age (years)	71.7 ± 5.5	72.2 ± 5.5	74.3 ± 6.4	73.2 ± 6.1	0.037
Gender (male, %)	49.1	38.7	32.4	46.5	0.140
Smoking (%)	8.9	10.2	12.1	10.0	0.102
DM duration (years)	11.1 ± 6.7	11.4 ± 7.3	14.3 ± 9.5	14.1 ± 7.7	0.003
BMI (kg/m^2^)	24.1 ± 3.4	25.7 ± 3.6	24.4 ± 4.2	26.0 ± 3.9	<0.001
FPG (mg/dL)	140.4 ± 31.4	142.7 ± 35.2	140.1 ± 44.3	141.1 ± 51.6	0.952
PPG (mg/dL)	190.1 ± 53.3	192.2 ± 58.4	230.0 ± 74.6	205.0 ± 71.1	0.008
HbA1c (%)	7.2 ± 1.3	7.1 ± 1.2	7.6 ± 1.4	7.5 ± 1.4	0.015
TC (mg/dL)	176.2 ± 31.9	165.6 ± 31.5	171.3 ± 27.4	170.6 ± 33.6	0.076
TG (mg/dL)	84.8 ± 30.6	120.3 ± 52.3	92.9 ± 34.1	134.2 ± 82.9	<0.001
LDL (mg/dL)	96.9 ± 25.1	94.9 ± 26.9	96.0 ± 26.7	96.9 ± 26.6	0.904
HDL (mg/dL)	58.5 ± 13.8	45.8 ± 12.5	56.5 ± 8.5	45.6 ± 14.9	<0.001
SBP (mmHg)	137.9 ± 16.6	141.2 ± 16.6	145.1 ± 19.4	146.7 ± 18.6	0.002
DBP (mmHg)	76.9 ± 9.8	76.3 ± 8.7	76.4 ± 10.3	78.6 ± 10.9	0.273
ACEI or ARB (%)	51.7	51.5	43.2	50.5	0.822
Cr (mg/dL)	0.9 ± 0.2	0.9 ± 0.3	1.0 ± 0.4	1.1 ± 0.4	<0.001
GPT (mg/dL)	22.5 ± 10.1	25.3 ± 13.6	24.3 ± 14.3	27.2 ± 16.0	0.107
eGFR (mL/min/1.73 m^2^)	80.9 ± 22.1	73.8 ± 22.2	68.4 ± 24.8	66.6 ± 22.5	<0.001
ACR (mg/g)	11.0 ± 7.2	10.0 ± 7.2	89.6 ± 55.0	101.0 ± 80.0	<0.001
CKD stage 3–4 (15 ≦ eGFR <60, %)	14.2	29.6	47.2	45.5	<0.001

Data are presented as the mean value ± standard deviation or %.

BMI = body mass index; FPG = fasting plasma glucose; PPG = post-prandial plasma glucose; HbA1c = glycosylated hemoglobin; TC = total cholesterol; TG = triglyceride; LDL = low-density lipoprotein cholesterol; HDL-C = high-density lipoprotein cholesterol; SBP = systolic blood pressure; DBP = diastolic blood pressure; GPT = glutamic-pyruvic transaminase; Cr = creatinine; eGFR = estimated glomerular filtration rate; ACR = urinary albumin-creatinine ratio; DKD = diabetic kidney disease.

### Urinary albumin-creatinine ratio and metabolic syndrome may predict worsening renal function

**Figure 2:**
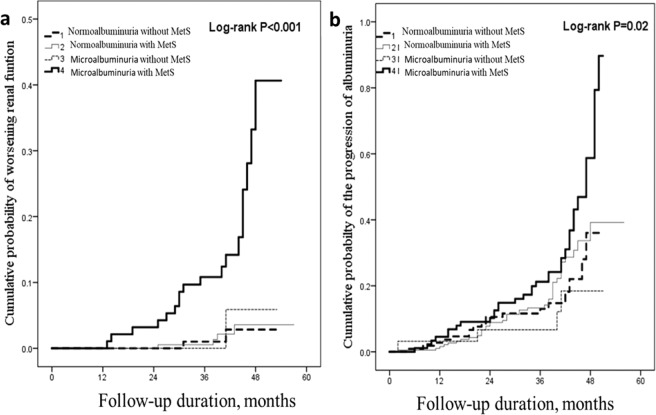
Kaplan-Meier estimates of probability of worsening renal function (panel a) or the progression of albuminuria (panel b) in elderly patients with T2DM according to urinary ACR and metabolic syndrome (MetS).

Of the 460 patients, 23 (5%) presented worsening renal function during the follow-up period. Cox regression analysis was used to explore the effect of metabolic syndrome and/or microalbuminuria on renal outcomes. In the univariable analysis, both microalbuminuria and metabolic syndrome were significantly associated with worsening renal function (Table 2, Model 1 and Model 2). After adjusting for confounding factors, both microalbuminuria (HR 5.42; 95% CI 2.23–13.17; P < 0.001) and metabolic syndrome (HR 3.61; 95% CI 1.07–12.10; P = 0.038) remained significantly associated with worsening renal function. As shown in Table 2, when patients were divided into four groups according to the presence of albuminuria and/or metabolic syndrome (Model 3), we observed that HRs gradually increased when these two factors were present. The group with both metabolic syndrome and microalbuminuria showed the highest cumulative rate of worsening renal function (HR 9.28; 95% CI 2.12–40.66; P = 0.003). We used the Kaplan-Meier method and the log-rank test to further analyze the data, and found a significant difference in the 5-year cumulative incidence of worsening renal function (Fig. 2a).

**Table 2:** Univariable and multivariable Cox proportional hazards models for worsening renal function and the progression of albuminuria in elderly patients with T2DM.

	Univariable		P	Multivariable		P
	HR 95% (CI)			HR 95% (CI)		
Worsening renal function^a^						
Model 1						
ACR < 30	1	Ref.		1	Ref.	
ACR > 30	7.10	(2.83–17.77)	<0.001	5.42	(2.23–13.17)	<0.001
Model 2						
Without MetS	1	Ref.		1	Ref.	
With MetS	3.54	(1.06–11.82)	0.040	3.61	(1.07–12.10)	0.038
Model 3						
ACR < 30 without MetS	1	Ref.		1	Ref.	
ACR < 30 with MetS	1.10	(0.20–6.02)	0.911	1.39	(0.28–7.38)	0.789
ACR > 30 without MetS	1.53	(0.14–16.93)	0.727	1.43	(0.13–15.40)	0.669
ACR > 30 with MetS	9.67	(2.24–41.68)	0.002	9.28	(2.12–40.66)	0.003
The progression of albuminuria^b^						
Model 1						
ACR < 30	1	Ref.		1	Ref.	
ACR > 30	1.40	(0.93–2.12)	0.108	1.37	(0.87–2.17)	0.174
Model 2						
Without MetS	1	Ref.		1	Ref.	
With MetS	1.66	(1.03–2.67)	0.037	1.60	(1.03–2.77)	0.039
Model 3						
ACR < 30 without MetS	1	Ref.		1	Ref.	
ACR < 30 with MetS	1.22	(0.70–2.12)	0.492	1.18	(0.67–2.08)	0.571
ACR > 30 without MetS	0.62	(0.21–1.83)	0.385	0.47	(0.14–1.62)	0.232
ACR > 30 with MetS	1.98	(1.11–3.51)	0.020	1.87	(1.01–3.47)	0.046

Model 1, vs. normoalbuminuria; model 2, vs. without metabolic syndrome; model 3, vs. normoalbuminuria without metabolic syndrome.

^a^Worsening renal function: adjusted for diabetes duration and HbA1c.

^b^Progression of albuminuria: adjusted for gender, obesity, diabetes duration, HbA1c, hypertension, dyslipidemia, baseline eGFR, ACEI or ARB use and CKD.

ACR = urinary albumin-creatinine ratio; MetS = metabolic syndrome.

### Metabolic syndrome promotes the progression of albuminuria

Of the 460 patients, 91 (19.8%) showed progression of albuminuria during follow-up. In the univariable analysis, metabolic syndrome, but not microalbuminuria, was significantly associated with the progression of albuminuria (Table 2, Model 1 and Model 2). After adjusting for other confounding factors, metabolic syndrome (HR 1.60; 95% CI 1.03–2.77; P = 0.039) was still significantly associated with albuminuria progression. As shown in Table 2, when patients were divided into four groups according to the presence of albuminuria and/or metabolic syndrome (Model 3), we observed that the group with both metabolic syndrome and microalbuminuria demonstrated the highest cumulative rate of albuminuria progression (HR 1.87; 95% CI 1.01–3.47; P = 0.046). We used the Kaplan-Meier method and the log-rank test for further analysis, and found a significant difference in the 5-year cumulative incidence of albuminuria progression (Fig. 2b).

### Effect of individual components of metabolic syndrome

To eliminate the effect of hypertension, we redefined metabolic syndrome by excluding the BP component (Table 3). Diabetic patients with two or three additional components, excluding BP and diabetes, were viewed as having metabolic syndrome. The resulting two groups with or without metabolic syndrome did not differ in SBP (142.9 ± 17.6 vs. 140.5 ± 15.9 mmHg). In the multivariable analysis, metabolic syndrome without the BP component could predict worsening renal function (HR 3.20; 95% CI 1.43–7.15; P = 0.005) but not albuminuria progression. When patients were divided into four groups according to the presence of microalbuminuria and/or metabolic syndrome without the BP component, the HR trend for worsening renal function was similar to the results for metabolic syndrome including the BP component, presented in Table 2. We further evaluated the effect of individual components and cumulative components of metabolic syndrome in elderly patients (see Supplementary Table S1). Regarding individual components, the BP component was significantly correlated with worsening renal function (HR 1.73; 95% CI 1.04–2.88; P = 0.034) and albuminuria progression (HR 1.31; 95% CI 1.02–1.70; P = 0.036) in the multivariable analysis. When we analyzed the cumulative components of metabolic syndrome, the presence of four components was not only correlated with worsening renal function (HR 10.07; 95% CI 1.61–62.97; P = 0.014) but also with the progression of albuminuria (HR 3.20; 95% CI 1.11–9.18; P = 0.031).

**Table 3:** Univariable and multivariable Cox proportional hazards models for worsening renal function and the progression of albuminuria in elderly patients with T2DM (metabolic syndrome without blood pressure component).

	Univariable		P	Multivariable		P
	HR 95% (CI)			HR 95% (CI)		
Worsening renal function^a^						
Model 1						
Without MetS	1	Ref.		1	Ref.	
With MetS	2.24	(1.02–4.91)	0.044	3.20	(1.43–7.15)	0.005
Model 2						
ACR < 30 without MetS	1	Ref.		1	Ref.	
ACR < 30 with MetS	3.27	(0.87–12.24)	0.492	1.99	(0.33–11.96)	0.454
ACR > 30 without MetS	6.10	(1.87–19.85)	0.003	5.18	(1.33–20.15)	0.018
ACR > 30 with MetS	9.49	(2.24–32.86)	<0.001	14.04	(3.70–53.30)	0.003
The progression of albuminuria^b^						
Model 1						
Without MetS	1	Ref.		1	Ref.	
With MetS	1.52	(1.021–2.29)	0.044	1.44	(0.922–2.25)	0.109
Model 2						
ACR < 30 without MetS	1	Ref.		1	Ref.	
ACR < 30 with MetS	1.06	(0.58–1.91)	0.840	1.06	(0.58–1.95)	0.828
ACR > 30 without MetS	1.10	(0.63–1.92)	0.724	0.86	(0.14–1.62)	0.232
ACR > 30 with MetS	2.38	(1.42–3.98)	0.001	1.87	(1.07–3.32)	0.027

Model 1, vs. without metabolic syndrome; model 2, vs. normoalbuminuria without metabolic syndrome.

^a^Worsening renal function: adjusted for diabetes duration and HbA1c.

^b^Progression of albuminuria: adjusted for gender, obesity, diabetes duration, HbA1c, hypertension, dyslipidemia, baseline eGFR, ACEI or ARB use and CKD.

ACR = urinary albumin-creatinine ratio; MetS = metabolic syndrome.

## Discussion

Multiple studies have reported that microalbuminuria is associated with a decline in renal function, ESRD, and adverse outcomes in CKD^20–22^. Furthermore, microalbuminuria can predict cardiovascular risk and total mortality in both diabetic and non-diabetic patients, as confirmed in several meta-analyses^23–27^. Microalbuminuria has also been associated with perivascular disease and stroke^28,29^. Even in patients with normoalbuminuria, the albuminuria level is still correlated with the development of microalbuminuria and proteinuria in both type 1 and type 2 diabetes, probably reflecting the early pathologic changes associated with diabetic nephropathy^30–33^. The prevalence of albuminuria increases with age^34,35^. In an elderly diabetic population, microalbuminuria was found to be a predictor of cardiovascular risk and mortality^34,36,37^. However, only few studies have evaluated whether albuminuria can predict renal outcomes in elderly patients with diabetes. In the elderly, an increased prevalence of albuminuria may be related to aging processes, such as glomerular and vascular sclerosis and tubular atrophy, and cannot be attributed only to diabetic nephropathy. A relatively older diabetes cohort comprised of 10,640 patients with a mean age of 66 years demonstrated that the risk of a renal event was higher in patients with albuminuria. Similar results were observed in our cohort, in which the mean age was 72 years. We found that the presence of albuminuria increased the HR for worsening renal function. However, there was no significant association for the progression of albuminuria. This could be due to the small sample size in addition to the heterogeneous causes of albuminuria in elderly patients; however, other parameters should be considered to distinguish the normal aging process from true diabetic nephropathy.

Some epidemiological studies have suggested an independent association between microalbuminuria and metabolic syndrome^13^. Some studies reported that the prevalence of microalbuminuria increases significantly as the number of metabolic syndrome components increases^13,38^. We observed a similar result in the current study, even though our cohort was older (Fig. 1). Metabolic syndrome also increases the risk of microalbuminuria. A Japanese study of subjects aged 40 to 87 years reported that the risk of microalbuminuria in patients with metabolic syndrome was increased by 1.99-fold after adjusting for age and gender compared to those without metabolic syndrome^39^. A Chinese population study found that metabolic syndrome increased the risk of microalbuminuria (OR 1.78; 95% CI 1.226–2.587) after adjusting for age and sex. For an elderly Chinese population, metabolic syndrome may be independently and closely associated with microalbuminuria^38^.

The prevalence of metabolic syndrome also increases with age, probably due to the age-related prevalence of abdominal obesity and insulin resistance^40,41^. Metabolic syndrome and insulin resistance can predict the risk and incidence of CKD in non-diabetic elderly patients^11^. However, the core pathological mechanism of T2DM is metabolic syndrome and obesity-related insulin resistance, which means that the age-related increase in metabolic syndrome may exacerbate renal dysfunction in elderly diabetic patients; however, this hypothesis has been little studied. We used elderly T2DM patients to assess the progression of renal disease and the presence of other components of metabolic syndrome besides diabetes. Indeed, metabolic syndrome in elderly patients not only increased the risk of worsening renal function, but also the transition to an advanced stage of albuminuria (Table 2, Model 2). As discussed earlier, the causes of albuminuria in aged individuals are complicated and cannot be attributed to diabetes alone. Therefore, using only albuminuria to predict renal disease progression in this group of patients may not be accurate. We also found that metabolic syndrome has an add-on effect for the prediction of renal disease progression (Table 2, Model 3). The HRs gradually increased when we divided the patients into four groups, indicating that the addition of metabolic syndrome to albuminuria provides a more precise risk stratification model for renal disease progression particularly in regard to worsening renal function.

Type 2 diabetes is often accompanied by hypertension, and the coexistence of both risk factors greatly accelerates the progression of DKD. Certainly, the BP component of metabolic syndrome may have affected the renal outcome in our patients. However, even when we excluded the effect of BP by redefining metabolic syndrome without the BP component, this risk stratification model using metabolic syndrome and albuminuria could still be successfully applied to predict worsening renal function and albuminuria progression (Table 3). However, considering only metabolic syndrome without the BP component cannot predict albuminuria progression. This is because the BP component itself may have a significant effect on albuminuria progression, as shown in Supplementary Table S1. When we excluded this component, the result was not significant in this small sample of patients. Indeed, most studies support a link between albuminuria and BP. It is generally accepted that the prevalence of albuminuria increases with the BP level. Although the criteria for the BP component in metabolic syndrome is defined as below 130/85 mmHg, this normal-high BP was still correlated with the prevalence of albuminuria^42,43^. Our study further demonstrated that normal-high BP promoted albuminuria progression in elderly patients. Further analysis of the cumulative components of metabolic syndrome indicated that as the number of components increased, the HRs for both worsening renal function and albuminuria progression increased. Therefore, it is reasonable that all components of metabolic syndrome may have independent effects on renal outcomes, and the significant effect of metabolic syndrome cannot be attributed to the BP component alone. These significant effects were especially predominant when we subsequently used both albuminuria and metabolic syndrome to predict renal outcomes.

To the best of our knowledge, this is the first study to stratify the risk for renal disease progression according to albuminuria and metabolic syndrome in elderly diabetic patients. However, our study has several limitations. Firstly, all patients enrolled in our study were from a single center, so our results have poor generalizability. Secondly, we corrected for the use of ACEI/ARB in the multivariable analysis, as these medications may have an effect on albuminuria. New medications that may influence albuminuria, such as SGLT2 inhibitors, were not evaluated in this study. Thirdly, our sample size was relatively small, which may influence the statistical power; therefore, some results may not have reached statistical significance. Lastly, we could not exclude other diseases that may worsen renal function and albuminuria progression, such as hypertensive nephrosclerosis and chronic glomerulonephritis.

In summary, this longitudinal observational study showed that metabolic syndrome may predict worsening renal function and progression to later stages of albuminuria in elderly TD2M patients. The addition of metabolic syndrome to albuminuria can improve risk stratification for renal disease progression in this group of patients.

## Supplementary information

Supplementary information.

## Data Availability

The datasets generated during and/or analysed during the current study are available from the corresponding author on reasonable request.
