# Supplementary material for: Addition of Metabolic Syndrome to Albuminuria Provides a New Risk Stratification Model for Diabetic Kidney Disease Progression in Elderly Patients
Source: Sci Rep. 2020 Apr 22;10:6788. doi: 10.1038/s41598-020-63967-9 (PMC7176677; doi:10.1038/s41598-020-63967-9)
Supplement: Supplementary file 1 — Supplementary information. [file 41598_2020_63967_MOESM1_ESM.docx]

**SUPPLEMENTARY INFORMATION**

**Addition of Metabolic Syndrome to Albuminuria Provides a New Risk Stratification Model for Diabetic Kidney Disease Progression in Elderly Patients.**

**Authors**: Hong-Mou Shih1,4, Shih-Ming Chuang2,3,*, Chun-Chuan Lee2, Sung-Chen Liu2, Ming-Chieh Tsai2,

**Author Affiliations:**

1 Division of Nephrology, Department of Internal Medicine, Mackay Memorial Hospital, Taipei, Taiwan

2 Division of Endocrinology and Metabolism, Department of Internal Medicine, Mackay Memorial Hospital, Taipei, Taiwan

3Mackay Junior College of Medicine, Nursing, and Management, Taipei, Taiwan

4Graduate Institute of Physiology, College of Medicine, National Taiwan University, Taipei, Taiwan

***Corresponding authors:** Shih-Ming Chuang, Division of Endocrinology and Metabolism, Department of Internal Medicine, Mackay Memorial Hospital, No. 92, Sec. 2, Zhongshan N. Rd., Taipei 10449, Taiwan, R.O.C

Telephone number:886-2-25433535, Fax number:886-25433533

E-mail address: gopacer@hotmail.com

| **Supplementary Table S1. Univariable and multivariable Cox proportional hazards models for worsening renal function and the progression of albuminuria with Individual components of the Metabolic Syndrome in elderly patients with T2DM** | | | | | | | | |
| --- | --- | --- | --- | --- | --- | --- | --- | --- |
|  |  | Univariable | |  |  | Multivariable | |  |
|  |  | HR 95% (CI) | | P |  | HR 95% (CI) | | P |
| Worsening renal function^a^ |  |  |  |  |  |  |  |  |
| Abdominal obesity |  | 0.86 | (0.59-1.26) | 0.449 |  | 0.95 | (0.59-1.54) | 0.844 |
| blood pressure > 130/85mmHg |  | 1.64 | (1.14-2.36) | 0.008 |  | 1.73 | (1.04-2.88) | 0.034 |
| HDL <40(men) or <50 mg/dL (women) |  | 0.85 | (0.52-1.38) | 0.508 |  | 1.44 | (0.85-2.43) | 0.177 |
| triglyceride >150 mg/dL |  | 1.13 | (0.76-1.67) | 0.551 |  | 0.83 | (0.49-1.43) | 0.502 |
| Metabolic syndrome^c^ |  |  |  |  |  |  |  |  |
| 2 components |  | 2.79 | (0.77-10.16) | 0.119 |  | 1.17 | (0.25-5.50) | 0.842 |
| 3 components |  | 3.44 | (0.86-13.75) | 0.081 |  | 3.45 | (0.65-18.39) | 0.146 |
| 4 components |  | 6.71 | (1.67-29.91) | 0.007 |  | 10.07 | (1.61-62.97) | 0.014 |
| The progression of albuminuria^b^ |  |  |  |  |  |  |  |  |
| Abdominal obesity |  | 1.01 | (0.84-1.22) | 0.907 |  | 1.19 | (0.84-1.67) | 0.578 |
| blood pressure > 130/85mmHg |  | 1.34 | (1.12-1.61) | 0.001 |  | 1.31 | (1.02-1.70) | 0.036 |
| HDL <40(men) or <50 mg/dL (women) |  | 0.84 | (0.66-1.08) | 0.170 |  | 1.10 | (0.79-1.53) | 0.587 |
| triglyceride >150 mg/dL |  | 1.33 | (1.12-1.58) | 0.001 |  | 1.09 | (0.81-1.47) | 0.578 |
| Metabolic syndrome^c^ |  |  |  |  |  |  |  |  |
| 2 components |  | 1.42 | (0.85-2.37) | 0.187 |  | 1.65 | (0.79-3.47) | 0.186 |
| 3 components |  | 1.48 | (0.82-2.68) | 0.193 |  | 1.76 | (0.69-4.47) | 0.237 |
| 4 components |  | 3.03 | (1.60-5.74)) | 0.001 |  | 3.20 | (1.11-9.18) | 0.031 |
| ^a^Worsening renal function: adjusted for diabetes duration and HbA1c  ^b^Progression of albuminuria: adjusted for gender, obesity, diabetes duration , HbA1c, hypertension, dyslipidemia, baseline eGFR, ACEI or ARB use and CKDDL= high density lipoprotein cholesterol  ^c^Comparsion to 0 or 1 component of metabolic syndrome except for diabetes | | | | | | | | |
